# Ultrasound-derived pelvic floor parameters and their association with functional impairment in gynecologic cancer survivors: a retrospective cohort study

**DOI:** 10.3389/fonc.2026.1856337

**Published:** 2026-06-11

**Authors:** Liping Zhang, Fang He, Sha Dou, Xiaoxiao Qiao, Wenyan Wen, Ruifang Yan, Yanping Li, Siying Jia, Lili Wang

**Affiliations:** Department of Obstetrics and Gynecology, Shanxi Bethune Hospital, Shanxi Academy of Medical Sciences, Tongji Shanxi Hospital, Third Hospital of Shanxi Medical University, Taiyuan, Shanxi, China

**Keywords:** functional outcomes, gynecologic cancer survivors, pelvic floor dysfunction, pelvic floor ultrasonography, pelvic organ descent, survivorship, ultrasound imaging parameters

## Abstract

**Background:**

Gynecologic cancer survivors often experience pelvic floor dysfunction (PFD). PFD is a common but underdiagnosed complication, likely as a result of structural and/or functional changes caused by treatments for their gynecologic cancers. Objective imaging assessments of pelvic floor integrity in this population are limited.

**Methods:**

This exploratory retrospective cohort study evaluated associations between pelvic floor ultrasonography parameters and patient-reported functional impairment in 150 survivors of cervical, endometrial, and ovarian cancer stratified by treatment modality. Pelvic floor muscle (PFM) thickness, mobility, and pelvic organ descent were assessed using standardized ultrasonography protocols. The validated Pelvic Floor Distress Inventory-20 (PFDI-20) was used to assess pelvic floor symptom burden and patient-reported functional impairment. Correlation and multivariable regression analyses were performed after adjustment for relevant clinical covariates. Receiver operating characteristic analysis evaluated the discriminatory capability of ultrasound-derived parameters.

**Results:**

Exposure to radiotherapy was associated with decreased thickness and mobility of the pelvic floor muscles (PFM), as well as increased pelvic organ descent (all P < 0.05). Survivors receiving radiotherapy had higher scores on the Pelvic Floor Distress Inventory 20 (PFDI-20) than those undergoing surgery without radiotherapy (P <0.001). Reduced thickness of the PFM (β = −0.42, P <0.001) and increases in descent of pelvic organs (β = 0.48, P <0.001) were independently associated with greater levels of functional impairment.

**Conclusion:**

The associations between ultrasound-derived pelvic floor parameters and self-reported functional impairments and symptom burden in gynecologic cancer survivors were significant. Additional prospective longitudinal studies are needed prior to recommending routine clinical implementation of these findings.

## 1 Introduction

Data for gynecologic malignancies (including cervical, endometrial, and ovarian) indicate that there is an increasing global burden on health care systems from these cancers due primarily to improvements in early detection and multimodal treatment strategies (1–3). With improving survival outcomes, greater attention has been directed toward long-term survivorship concerns, particularly treatment-associated functional impairments and their impact on physical functioning, symptom burden, and survivorship-related functioning (4, 5). Within this area of long-term survivorship, pelvic floor dysfunction (PFD), which is commonly under-appreciated as a complication of gynecologic cancer survivorship, is a frequent occurrence in this population of survivors (6, 7).

PFD is defined as a collection of clinical conditions that disrupt the normal function of the pelvic floor, including urinary incontinence, pelvic organ prolapse, and colorectal dysfunction, and the impact of these conditions can affect every aspect of daily living and range from negative impacts in daily functioning to negative impacts in psychosocial functioning (8–10). The etiology of PFD in oncologic populations is complex, with surgical injury disrupting pelvic support structures, radiation-induced fibrosis, and injury to the nervous and muscular systems occurring together and contributing to PFD in this population (11–13). Specifically, the effects of radiation therapy on the tissue progress through stages of the tissue remodeling process naturally after therapy and result in progressive loss of elastic tissue, impaired cellular circulation, and delivery of oxygen to the pelvic supportive tissues, all of which are thought to lead to loss of pelvic floor function (14, 15).

Though the impact of PFD is clinically significant, traditional approaches to assessing PFD have relied heavily upon patient-reported outcomes and subjective evaluation, both of which do not adequately represent the structure and function of the pelvic floor in patients diagnosed with cancers (16). Accordingly, there is growing interest in objective, reproducible, and non-invasive imaging approaches capable of evaluating pelvic floor integrity during survivorship follow-up.

In assessing pelvic floor integrity, pelvic floor ultrasound has emerged as a novel imaging modality capable of dynamic assessment of pelvic floor anatomy and function in real time (17). Pelvic floor ultrasound is advantageous because it is generally accessible and low-cost, and it allows quantification of parameters such as muscle thickness, pelvic floor mobility, and pelvic organ descent. However, while this technology is gaining traction for use in routine gynecologic practice, the evaluation of ultrasound as a quantitative imaging modality within this patient population is not yet well established.

The connections between pelvic floor ultrasound compositions, as defined by ultrasound, and patient functional impairments following treatment of gynecological cancer are not fully understood. It is not yet clear whether there are any clinically significant imaging findings to correlate with the survivorship-related dysfunction of the pelvic floor.

Thus, the aims of this analysis are to evaluate the associations between pelvic floor ultrasound composition and patients’ functional impairment status following various treatment modalities for gynecological cancers. The analysis of the associations between imaging and functional impairments is done retrospectively. It has thus been designed as an exploratory analysis of imaging functions related to gynecological cancer survivorship, to produce clinically relevant signal data that could form the basis for future prospective studies.

## 2 Methods

### 2.1 Study design and setting

The present study was designed as an exploratory retrospective cohort analysis conducted in the Department of Gynecologic Oncology at a tertiary oncology facility. Patients who received care between January 2020 and December 2023 were screened for eligibility. This study followed the STROBE (Strengthening the Reporting of Observational Studies in Epidemiology) reporting guideline for observational studies to improve methodological transparency and reporting quality. Eligible patients were identified from the institutional electronic medical records and included in the order of their listing to minimize selection bias. Data collection occurred as part of routine clinical follow-up and reflected real-world clinical practice.

### 2.2 Study population

The female participants in this study all survived gynecologic cancers (cervical, endometrial, and ovarian) and had completed their initial cancer treatments. The minimum eligibility age was 18. All groups of women had a histologically confirmed gynecologic cancer and had completed their last cancer treatment at least 6 months before evaluation, to allow for healing and return of some degree of normal function following cancer treatment. To be included in final analyses, every participant must have been available for assessment of his/her clinical status, imaging tests, and functional tests.

Women were excluded from this study if they had an active or recurrent malignancy, had a surgical resection of the pelvis within the previous six months, or had neurological conditions that independently affected pelvic floor function. Also, participants with incomplete imaging or questionnaire data were excluded from the final analyses to improve analytical consistency.

Data on detailed treatment variables (e.g., cancer stage, total amount of radiation exposure, type of surgery) were not available for all participants at the time of the study. Other potential confounders, specifically variables related to patients’ baseline demographic characteristics and selection bias due to the retrospective nature of the study, cannot be thoroughly assessed.

### 2.3 Treatment-based stratification

To assess how different treatment types affect the strength and shape of the pelvic floor structure, participants were divided into the following groups: Group A, patients who underwent surgery only; Group B, patients who underwent surgery followed by radiotherapy; and Group C, patients who received radiotherapy only.

### 2.4 Pelvic floor ultrasonography protocol

A high-resolution ultrasound machine (GE Logiq P9) was used to conduct pelvic floor ultrasound with standardized evaluation methods. Pelvic floor ultrasound was done through the perineum using a curved array transducer at 3.5 - 5.0 MHz in the midsagittal plane. The same sonographer, known to be proficient in pelvic floor ultrasound, completed all examinations to limit inter-operator variability.

The participants were placed in the supine lithotomy position with a standardized bladder volume (about 150–200 mL) before images were acquired. Anatomical landmarks included the pubic bone, bladder neck, anorectal junction, and puborectalis muscle.

For standardizing imaging, pelvic floor measurements were done in three different conditions: (1) rest, (2) maximal voluntary pelvic floor contraction, and (3) during forced expiration via the Valsalva maneuver. The thickness of the pelvic floor muscles was determined using fixed anatomical reference points at rest and during contraction. Pelvic floor mobility was assessed by the displacement of the puborectalis muscle between resting and contraction states. Pelvic organ descent was defined as the maximal inferior displacement relative to the inferoposterior margin of the symphysis pubis during the Valsalva maneuver.

All measurements were performed following a standardized imaging protocol; each parameter was measured 3 times to reduce random measurement variability. Detailed methodological descriptions were provided to improve reproducibility and facilitate future validation studies. Representative ultrasound images were not included because the quality and standardization of retrospective image archives were inconsistent across examinations.

### 2.5 Outcome measures

Pelvic floor functional impairment was evaluated using the validated Chinese version of the Pelvic Floor Distress Inventory-20 (PFDI-20). The PFDI-20 can assess symptom burden across three areas (urinary, colorectal, and prolapse) and provide a single score that represents the severity of pelvic floor dysfunction.

Other outcome measures included symptom severity indices and patient-reported measures of functional impairment related to pelvic floor dysfunction. These measures were collected by a clinical staff member (with training) during their normal follow-up visits to promote consistency and reliability of the data collection process.

### 2.6 Data collection and covariates

Demographic and clinical characteristics were taken from the hospitals’ medical record systems. Age, BMI, parity, cancer diagnosis, and treatment were recorded as variables. These variables were chosen because they may affect pelvic floor function and will therefore be analyzed as covariates in the statistical analysis to account for potential confounding. The data were coded/anonymized prior to being transferred to the researcher for analysis to maintain patient confidentiality.

### 2.7 Statistical analysis

SPSS version 26.0 (IBM Corporation, Armonk, NY, USA) was used to perform all statistical analyses. Continuous variables were presented as mean ± standard deviation, while categorical variables were summarized as frequencies and percentages.

The normal distribution of data was confirmed using the Shapiro-Wilk test, and the assumption of equal variances was assessed using Levene’s test. One-way analysis of variance (ANOVA) was used to compare the means of continuous variables between treatment groups, and chi-square tests were used to compare categorical variables. When the overall ANOVA was significant, Tukey’s *post hoc* multiple comparisons test was also performed to compare each pair of treatment group means. In instances where parametric assumptions were violated, equivalent nonparametric tests were used when appropriate.

Associations between ultrasound-derived pelvic floor parameters and functional outcomes were assessed using Pearson correlation analysis. Multivariable linear regression analysis was performed to identify variables independently associated with functional impairment after adjustment for age, BMI, parity, and treatment modality. Because of the retrospective observational design and cross-sectional assessment of imaging and symptom outcomes, all regression findings were interpreted as associations rather than causal relationships.

The ability of ultrasound parameters to differentiate survivors with high functional impairment was evaluated using receiver operating characteristic (ROC) curve analysis. High levels of functional impairment were defined as having a pelvic floor dysfunction inventory PFDI-20 score greater than the cohort median, as no established threshold currently exists for women with gynecologic cancer. The area under the ROC curve (AUC) was used to determine the discriminatory accuracy.

Given that the group receiving radiation therapy as the sole treatment was small relative to the other treatment groups, any findings from this group were interpreted with great caution and considered exploratory. A two-tailed P-value < 0.05 was judged to be statistically significant.

### 2.8 Ethical considerations

The Institutional Review Board approved this retrospective study (Approval No. YXLL-2024-062). The requirement for written informed consent was waived because de-identified retrospective clinical data were analyzed. All data were de-identified before analysis to protect patient confidentiality.

## 3 Results

### 3.1 Study population and baseline characteristics

**Figure 1:**
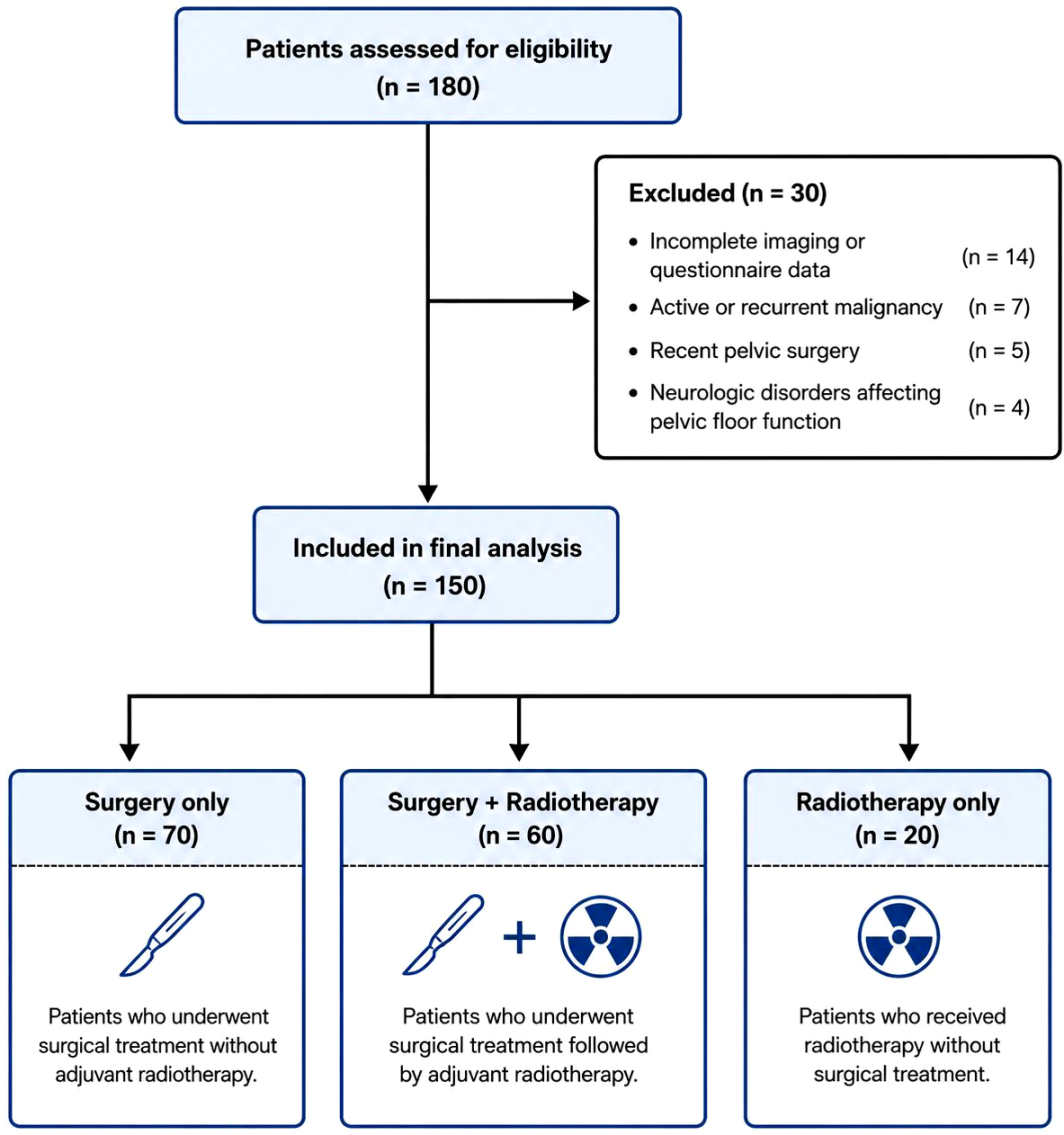
Study flow diagram of patient selection and cohort stratification. The flow diagram illustrates patient screening, eligibility assessment, exclusion criteria, and treatment-based stratification of gynecologic cancer survivors included in the final analysis. A total of 180 patients were assessed for eligibility, of whom 30 were excluded because of incomplete imaging/questionnaire data or failure to meet inclusion criteria. The final study cohort (n = 150) was stratified into three groups: surgery only (n = 70), surgery combined with radiotherapy (n = 60), and radiotherapy only (n = 20).

Initially, 180 patients were assessed for eligibility; however, 30 were excluded due to missing data or failure to meet the inclusion criteria. Ultimately, there were 150 eligible survivors with gynecologic cancer. The survivors were assigned to 3 treatment groups according to their original treatment: Surgery only (n = 70); Surgery + Radiotherapy (n = 60); or Radiotherapy only (n = 20), as shown in **Figure 1**.

**Table 1** provides the characteristics of the participants in terms of demographics and clinical at baseline. Categorical values are provided as absolute counts as well as percentages (n%). When comparing groups by age (P = 0.312), BMI (P = 0.278), parity (P = 0.221), and cancer type (P = 0.145), we found that none of the groups differed significantly in baseline characteristics.

**Table 1:** Baseline characteristics.

Variable	Total(n=150)	Surgery only (n=70)	Surgery + RT (N = 60)	RT only (N = 20)	P-value
Age(years)	42.3 ± 8.7	41.5 ± 8.2	43.6 ± 9.1	42.9 ± 8.5	0.312
BMI(KG/M^2^)	26.4 ± 4.2	25.8 ± 4.0	27.1 ± 4.5	26.7 ± 4.1	0.278
Parity	2.5 ± 1.3	2.3 ± 1.2	2.7 ± 1.4	2.6 ± 1.3	0.221
Cervical cancer, n (%)	68 (45.3%)	28 (40.0%)	31 (51.7%)	9 (45.0%)	
Endometrial cancer, n (%)	52 (34.7%)	27 (38.6%)	18 (30.0%)	7 (35.0%)	
Ovarian cancer, n (%)	30 (20.0%)	15 (21.4%)	11 (18.3%)	4 (20.0%)	0.145
Time since treatment (months)	14.2 ± 5.3	13.8 ± 5.1	14.6 ± 5.6	14.1 ± 5.2	0.564

Data are presented as mean ± standard deviation (SD) or n (%), as appropriate. P-values were calculated using one-way analysis of variance (ANOVA) for continuous variables and chi-square tests for categorical variables. BMI, body mass index; RT, radiotherapy.

Although baseline demographics were generally balanced across groups, caution is warranted when interpreting results, as residual confounding cannot be completely eliminated in a retrospective observational study. Additionally, caution should be exercised when interpreting findings for the subgroup of radiotherapy-only participants due to their relatively small sample size.

### 3.2 Ultrasound-derived pelvic floor parameters

**Figure 2:**
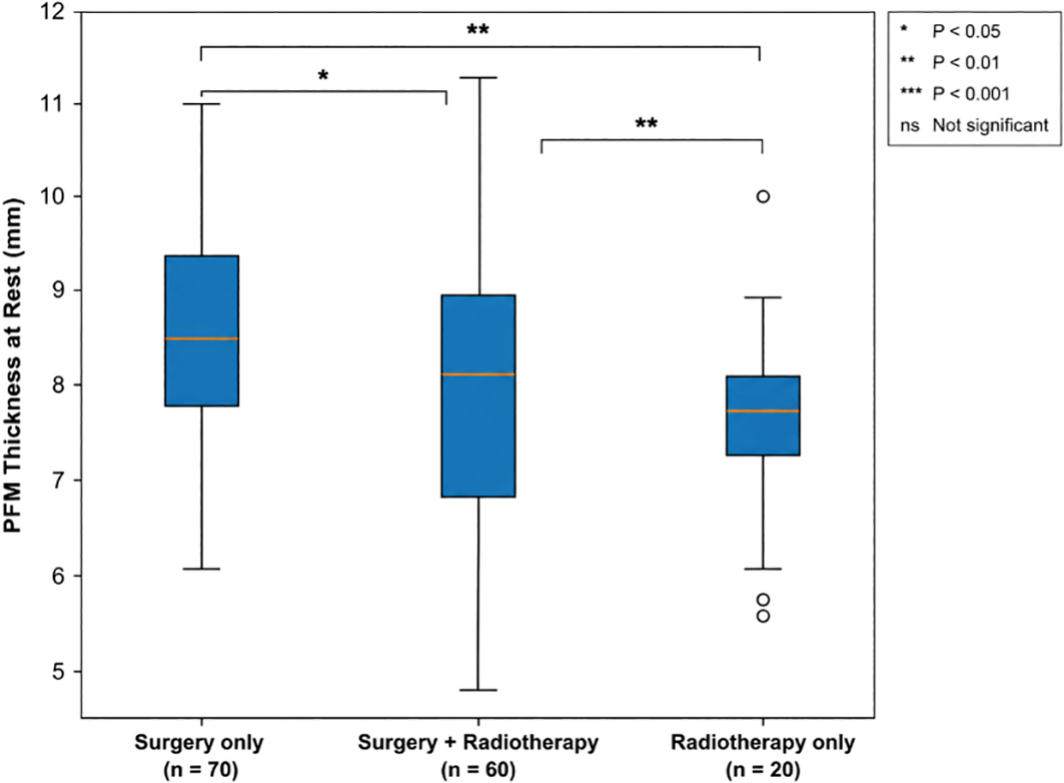
Comparison of ultrasound-derived pelvic floor parameters across treatment groups. Box-and-whisker plots demonstrate differences in pelvic floor muscle thickness among treatment groups. Boxes represent the interquartile range, horizontal lines indicate median values, and whiskers represent variability outside the upper and lower quartiles. Statistical comparisons were performed using one-way ANOVA with Tukey’s *post-hoc* test. *P < 0.05; **P < 0.01.

**Table 2** shows comparative analyses across all ultrasound-derived pelvic floor (PFM) parameters by treatment group; **Figure 2** displays the association of ultrasound-derived pelvic floor parameters by treatment group. All ultrasound-derived pelvic floor muscle (PFM) parameters showed statistically significant differences between treatment groups.

**Table 2:** Comparison of ultrasound-derived pelvic floor parameters according to treatment modality.

Parameter	Surgery only (n=70)	Surgery + RT (n=60)	RT only (n=20)	P-value
PFM thickness at rest (mm)	8.6 ± 1.2	7.9 ± 1.3^a^	7.8 ± 1.4^a^	0.021
PFM thickness during contraction (mm)	13.1 ± 1.4	12.2 ± 1.6^a^	12.0 ± 1.5^a^	0.018
Pelvic organ descent (mm)	14.8 ± 2.9	17.5 ± 3.3^a^	18.1 ± 3.5^a^	<0.001
PFM mobility (mm)	5.6 ± 0.9	4.8 ± 1.0^a^	4.6 ± 1.1^a^	0.009

Data are presented as mean ± standard deviation (SD). P-values were obtained using one-way analysis of variance (ANOVA). Pairwise comparisons were assessed using Tukey’s *post hoc* test. Superscript “a” indicates statistically significant difference compared with the surgery-only group (P < 0.05). PFM, pelvic floor muscle; RT, radiotherapy.

Patients who received combined surgery and radiotherapy (7.9 ± 1.3 mm) and/or radiotherapy only (7.8 ± 1.4 mm) had significantly reduced resting PFM thickness compared to those who received surgery alone (8.6 ± 1.2 mm; P = 0.021). Similarly, patients receiving both combined surgery and radiotherapy treatment had significantly reduced PFM thickness during contraction (P = 0.018).

Pelvic organ descent was markedly increased in patients who had received radiotherapy, with the highest descent observed among those receiving radiotherapy only (18.1 ± 3.5 mm), followed by that for surgery and radiotherapy (17.5 ± 3.3 mm), and least of all for those undergoing surgery only (14.8 ± 2.9 mm; P < 0.001).

Radiotherapy patients exhibited significantly decreased PFM (P = 0.009) motion, indicating impaired dynamic pelvic floor function.

Overall, radiotherapy exposure was associated with less favorable ultrasound-derived pelvic floor parameters, including both structural support and dynamic functional measurements. Pairwise group comparisons were additionally assessed using Tukey’s *post-hoc* analysis, as presented in **Table 2**.

### 3.3 Functional outcomes and symptom burden

**Table 3** presents the summary of functional outcomes from the study. Statistically significant differences were found in pelvic floor dysfunction between the different treatment groups.

**Table 3:** Functional outcomes and symptom burden according to treatment modality.

Outcome	Surgery only (n=70)	Surgery + RT (n=60)	RT only (n=20)	P-value
PFDI-20 total	62.4 ± 7.8	71.6 ± 8.5^a^	74.2 ± 9.1^a^	<0.001
Urinary subscale score	21.3 ± 4.5	25.8 ± 5.1^a^	27.0 ± 5.6^a^	0.002
Colorectal subscale score	18.5 ± 3.9	21.7 ± 4.3^a^	22.4 ± 4.8^a^	0.006
Prolapse subscale score	22.6 ± 4.8	24.1 ± 5.0	24.8 ± 5.3	0.081
Functional symptom	2.1 ± 0.6	2.8 ± 0.7^a^	3.0 ± 0.8^a^	<0.001
burden score				

Data are presented as mean ± standard deviation (SD). P-values were calculated using one-way analysis of variance (ANOVA). Pairwise comparisons were assessed using Tukey’s *post hoc* test. Superscript “a” indicates statistically significant difference compared with the surgery-only group (P < 0.05). PFDI-20, Pelvic Floor Distress Inventory-20; RT, radiotherapy.

Pelvic Floor Distress Inventory-20 (PFDI-20) mean total scores were significantly higher for radiotherapy-only patients (74.2 ± 9.1) than for surgery-alone patients (62.4 ± 7.8; P < 0.001). Patients receiving combined treatment were also worse off than those receiving surgery alone (71.6 ± 8.5).

Urinary and colorectal symptom scores were higher in both groups treated with radiotherapy than in those who had received surgery (P = 0.002 for urinary; P = 0.006 for colorectal). The prolapse subscale scores were higher with radiotherapy than with surgery alone, but the difference did not reach statistical significance (P = 0.081).

Patients exposed to radiotherapy demonstrated greater symptom burden and worse patient-reported functional impairment compared with patients treated with surgery alone.

### 3.4 Correlation between ultrasound parameters and functional outcomes

**Figure 3:**
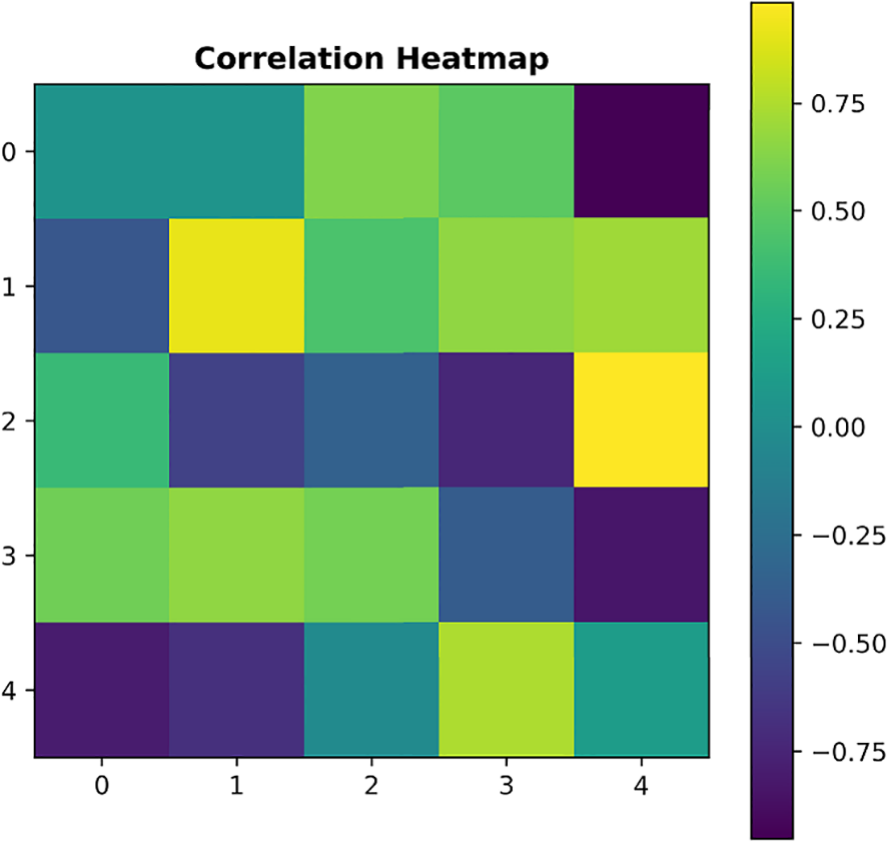
Correlation heatmap demonstrating associations between ultrasound-derived pelvic floor parameters and functional outcomes. The heatmap illustrates the strength and direction of correlations between ultrasound-derived pelvic floor parameters and patient-reported functional impairment measures. Pelvic floor muscle (PFM) thickness and mobility were negatively correlated with PFDI-20 scores, whereas pelvic organ descent was positively correlated with symptom burden and functional impairment. Color intensity reflects the magnitude of correlation coefficients, with warmer colors indicating stronger positive correlations and cooler colors indicating stronger negative correlations.

**Figure 3** shows the connections between ultrasound-derived parameters and functional outcomes. Regarding pelvic floor muscle thickness, there was a negative correlation with PFDI-20 scores; thus, reduced pelvic floor muscle thickness is associated with greater functional impairment.

In contrast, pelvic organ descent also had a positive correlation with functional impairment, so the more anatomic displacement there is, the more likely a patient is to experience worsening symptoms.

Also, pelvic floor muscle (PFM) mobility was negatively correlated with symptom severity, indicating that dynamic muscle function is necessary to support pelvic floor integrity.

Quantitative correlation analyses, including correlation coefficients (r values), 95% confidence intervals (CI), and corresponding P-values, are summarized in **Table 4** to improve statistical transparency and reproducibility.

**Table 4:** ROC analysis of ultrasound parameters for predicting functional impairment.

Ultrasound parameter	AUC (95%Cl)	Cutoff value	Sensitivity (%)	Specificity (%)	Youden index	P-value
PFM thickness at rest	0.76 (0.68-0.84)	<8.1mm	72.0	69.0	0.41	<0.001
PFM thickness during contraction	0.73 (0.65-0.81)	<12.4mm	70.0	66.0	0.36	<0.001
Pelvic organ descent	0.82 (0.75-0.89)	>16.5mm	81.0	74.0	0.55	<0.001
PFM mobility	0.70 (0.62–0.78)	<5.0mm	65.0	64.0	0.29	<0.002

ROC analysis was performed to evaluate the discriminatory capability of ultrasound-derived pelvic floor parameters for identifying survivors with higher functional impairment. High functional impairment was defined as a PFDI-20 score above the cohort median value for exploratory analysis. AUC, area under the curve; CI, confidence interval; PFM, pelvic floor muscle.

The overall observed relationships are clinically relevant, as patients’ functional impairment is associated with pelvic floor measurements obtained by ultrasound.

### 3.5 Multivariable regression analysis

Multivariable linear regression was performed to determine which independent variables were related to pelvic floor dysfunction. The findings are shown in **Table 5** above.

**Table 5:** Multivariable linear regression analysis of factors associated with functional impairment.

Variable	*β*	95% Cl	P-value
PFM thickness at rest	-0.42	-0.58 to -0.26	<0.001
PFM thickness during contraction	-0.35	-0.51 to -0.19	0.002
Pelvic organ descent	0.48	0.30 to 0.66	<0.001
PFM mobility	-0.21	-0.39 to -0.003	0.024
Age	0.09	-0.05 to 0.23	0.211
BMI	0.12	-0.03 to 0.27	0.118
Parity	0.08	-0.06 to 0.22	0.267
Treatment (Surgery + RT)	0.37	0.18 to 0.56	<0.001

Multivariable linear regression analysis adjusted for age, body mass index (BMI), parity, and treatment modality. β values represent standardized regression coefficients. CI, confidence interval. P < 0.05 indicates statistical significance. PFM, pelvic floor muscle; RT, radiotherapy.

The presence of pelvic organ descent was most strongly associated with functional disability (β = 0.48, 95% CI: 0.30 to 0.66, P < 0.001). In addition to this, PFM thickness at rest (β = −0.42, P < 0.001) and PFM thickness during contraction (β = −0.35, P = 0.002) were both found to be associated with a reduced risk of functional disability; these associations suggest that both of these independent variables may be protective factors.

PFM mobility was also determined to significantly correlate with better functional outcomes (β = -021; P = 0.024). Among clinical variables, combined surgical and radiation treatment was a statistically significant independent factor associated with poorer functional outcomes (β = 0.37, P < 0.001).

Age, body mass index (BMI), and number of children delivered were not statistically significantly associated with functional disability in the final adjusted model.

Although the observed associations were directionally consistent across analyses, interpretation should remain cautious given the retrospective observational design and the possibility of residual confounding.

### 3.6 Discriminatory capability of ultrasound parameters

**Figure 4:**
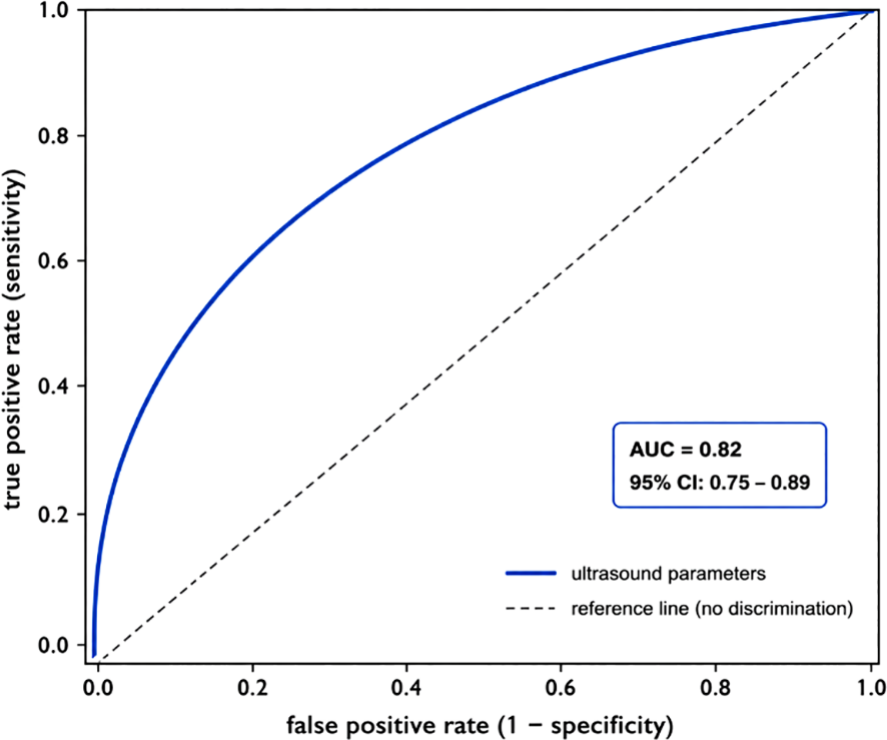
Receiver operating characteristic (ROC) curve demonstrating the discriminatory capability of ultrasound-derived pelvic floor parameters for functional impairment. Receiver operating characteristic (ROC) curve illustrating the discriminatory capability of ultrasound-derived pelvic floor parameters for differentiating survivors with higher functional impairment, defined according to PFDI-20 scores above the cohort median value. The area under the curve (AUC) demonstrated moderate discriminatory performance (AUC = 0.82; 95% CI: 0.75–0.89). The dashed diagonal line represents the reference distribution.

The ability of ultrasound-derived parameters to differentiate patients with high versus low functional impairment was assessed using receiver operating characteristic (ROC) curve analysis (**Figure 4**).

We defined high functional impairment in this study as PFDI-20 scores greater than the median of this cohort for performing exploratory ROC analysis, since there are currently no validated thresholds for survivors of gynecological cancer.

Pelvic organ descent had the greatest ability to discriminate patients based on their degree of dysfunction (AUC = 0.82, 95% CI: 0.75-0.89) when compared with PFM thickness at rest (AUC = 0.76), PFM thickness while contracting (AUC = 0.73), and PFM mobility (AUC = 0.70).

Detailed ROC performance metrics, including cut-off values, sensitivity, specificity, and Youden index, are summarized in **Table 4**.

Overall, ultrasound-based pelvic floor metrics were found to have moderate discriminatory power in separating survivors with higher levels of symptoms and greater functional impairment.

## 4 Discussion

### 4.1 Principal findings

This research utilized a retrospective cohort design to retrospectively analyze ultrasound-derived pelvic floor metrics and their relationships to self-reported functional impairment and symptom burden among gynecologic cancer patients. Specifically, lower pelvic floor muscle (PFM) thickness and mobility, and higher pelvic organ drop, were associated with poor functional outcomes across treatment modalities. These objective ultrasound findings support clinically significant links between ultrasound-defined pelvic floor findings and survivorship-related pelvic floor dysfunction.

Associations also remained directionally consistent after adjustment for clinically important covariates, suggesting that ultrasound-derived pelvic floor metrics could provide clinically relevant imaging correlates of survivorship-related pelvic floor dysfunction (18–22). Because ultrasound assessments and symptom reporting were done at a single time point, causal associations cannot be inferred.

Additional evidence exists for treatment modality differences contributing to pelvic floor structure and function. Patients treated with radiotherapy (either alone or in conjunction with surgical intervention) had less favorable pelvic floor ultrasound measurements and greater symptom burden than patients treated with surgical intervention alone. These findings generally reinforce prior literature documenting the long-term effects of radiotherapy exposure on pelvic tissue alterations, including the development of fibrosis, vascular compromise, and/or muscular and neurological dysfunction (23–25).

### 4.2 Interpretation of imaging–function relationships

In a survivorship population of gynecologic cancer survivors, an inverse relationship between PFM thickness and functional limitations indicates that PFM integrity may support pelvic floor function. Other studies have also found decreased PFM support to be associated with increased symptoms of urinary incontinence and prolapse after pelvic surgery (26–28).

Decreased PFM mobility is associated with greater symptom burden and is thought to reflect poor neuromuscular coordination and reduced elasticity of pelvic tissues. Studies conducted on both cancer and non-cancer survivors have found evidence of neuromuscular problems and tissue fibrosis leading to impaired dynamic pelvic floor support (29–32).

In the adjusted regression models, pelvic organ descent displayed the strongest independent association with functional impairments. This finding corroborates the results of imaging studies (previously cited) that have demonstrated that displacement of anatomical structures is related to the severity of certain pelvic floor symptoms (33–36). However, these findings should be interpreted with caution due to potential biases arising from a retrospective observational design and the simultaneous assessment of function and imaging.

### 4.3 Impact of treatment modality

Patients who received combined treatment with radiotherapy (XRT) exhibited the largest differences in pelvic floor ultrasound variables and functional impairment scores. This implies that patients receiving multimodal oncologic therapies in the survivorship follow-up may experience more pronounced pelvic-floor dysfunction than those who received only one type of therapy (surgery or XRT).

Radiotherapy was associated with decreased pelvic floor muscle thickness, reduced motion, and increased pelvic organ descent. Previous publications have also shown an association between pelvic radiotherapy and long-term remodeling of pelvic tissue, fibrosis, injury to vascular structures, and neuromuscular impairment (37, 38). Since granular data on radiation types and doses were not consistently available, caution is warranted when interpreting the current findings as definitive evidence of radiation-specific direct causality.

Patients who underwent surgery alone had evidence of preserved pelvic floor structure and function. Although the findings may have implications for survivorship, they are not yet recommended as the basis for clinical decisions based on routine imaging.

### 4.4 Clinical implications

The conclusion of this research has significant implications for the management of women who survive gynecologic cancer. Pelvic floor ultrasound is a low-cost, easy way to identify and quantify structural/functional problems in the female pelvic floor. Ultrasound provides an objective way to quantify factors that can inform clinical decisions, rather than relying solely on subjective measures of the problem (39, 40).

The observed associations between ultrasound parameters and functional outcomes, as demonstrated by ROC analysis, suggest that these ultrasound measures. These findings suggest that ultrasound-derived parameters may reflect clinically relevant structural and functional alterations associated with survivorship-related pelvic floor dysfunction. Ultimately, this could allow for early intervention through systematic pelvic floor rehabilitation, which has been shown to improve clinical outcomes for women with pelvic floor dysfunction (41).

Furthermore, combining imaging with self-reported measures of a woman’s status provides a more holistic understanding of her overall status, enabling a more individualized and multidisciplinary approach to survivorship care (42).

### 4.5 Comparison with existing literature

Pelvic floor dysfunction has been recognized as a common side effect of gynecologic cancer treatment by earlier studies, but many of those studies have mostly relied on the use of subjective symptom measurement tools. This study builds on prior literature by incorporating objective imaging measurement tools and demonstrating a relationship between their use and functional outcome measures (43).

Compared with previous studies, this study offers a broader scope of evaluation by integrating structural, functional, and patient-reported data into a single analytic model. The use of treatment-based stratification and multivariable adjustment methods together will yield a more robust set of findings on the mechanisms underlying the development of pelvic floor dysfunction.

### 4.6 Strengths of the study

Despite the limitations of this study, it has several significant strengths. First, all participants in this study were required to have pelvic floor ultrasonography acquired using a standardized protocol and imaging methods. Second, the use of ultrasound parameters alongside self-reported outcomes provides a multidimensional clinical picture that integrates ultrasonographic and self-reported outcome information. Finally, the treatment-based analysis provides a real-world example of the relationship between pelvic floor structural changes and symptom burden in patients with gynecological oncology.

Additional strengths include the use of standardized outcome measures, predefined imaging acquisition protocols, and multivariable regression analyses to partially account for potential confounding variables.

### 4.7 Limitations

There are several significant limitations that should be identified. First, the retrospective, single-center study design may increase the risk of selection bias, making generalization to the broader population of gynecologic oncology patients more difficult. The single-center design of the study may limit generalizability to larger populations of gynecologic oncology patients and to settings that provide survivorship care.

Second, there was no way to obtain baseline pelvic floor function prior to the start of oncologic treatment; therefore, assessing pre-treatment functional status is limited. Third, the small number of patients in the radiotherapy-only subgroup could decrease the statistical power of any subgroup-specific analyses. Fourth, significant oncologic variables (tumor stage, radiation dose, radiation field, menopausal status, hormonal status, method of delivery, history of rehabilitation, etc.) could not be included in the adjusted analyses due to insufficient data.

Residual confounding cannot necessarily be ruled out from oncologic treatment selection and baseline patient characteristics. In addition, formal intra- and inter-observer reliability testing using intraclass correlation coefficients (ICC) was not performed because of the retrospective design; however, all ultrasound evaluations were conducted by the same experienced sonographer using a predefined standardized imaging protocol to minimize measurement variability.

Finally, the assessments using ultrasound and symptoms were conducted at a single point in time (cross-sectional), and therefore, temporal relationships and causality cannot be established. Future studies should use multicenter, longitudinal, prospective designs to validate these findings and establish temporal relationships between ultrasonographic pelvic floor parameters and functional impairment associated with survivorship.

### 4.8 Future directions

Future long-term studies that compare standardized cancer treatments with measures of radiation exposure and baseline pelvic floor assessments are also needed to better support these findings. Newer imaging techniques, such as three-dimensional ultrasound and automated image analysis, may allow for improved reproducibility of pelvic floor assessments and enable quantitative evaluation of pelvic floor dysfunction among cancer survivors.

Overall, the ultrasound-derived pelvic floor parameters measured in the current study suggest there is an association between patient-reported functional impairment and symptom burden in gynecological cancer survivors. Therefore, additional long-term assessments are warranted prior to incorporation into gynecological cancer survivorship care programs.

## 5 Conclusion

In conclusion, this research confirmed that ultrasound information for the pelvic floor was associated with the individual experience of functional difficulties as well as how symptoms impacted their daily lives (Symptom Burden), among gynecological cancer survivors. While decreased pelvic floor muscle volume and movement, and increased pelvic organ descent were independently associated with general symptom burden and functional difficulty across all treatment modalities, patients received a greater burden of symptoms and functional impacts with the combination of surgery and/or radiation compared to surgery alone. Also, as these were retrospective and cross-sectional in design, the conclusions drawn from this study cannot be interpreted as establishing causality.

Ultrasound evaluation of the pelvic floor may provide an alternative means of evaluating survivorship-related pelvic floor dysfunction in a clinically relevant manner. Additionally, pelvic floor ultrasound may complement or verify prognostic findings in this unique population by assessing functional impairment; however, further prospective longitudinal studies are needed prior to recommending routine clinical implementation.

Finally, future multicenter prospective longitudinal studies with standardized oncologic and imaging measurements are needed to confirm these results and clarify the role of pelvic floor ultrasound in assessing survivorship status in patients with gynecological cancer.

## Data Availability

The datasets presented in this study can be found in online repositories. The names of the repository/repositories and accession number(s) can be found in the article/supplementary material.
